# Protective Effect of Curcuma Extract in an *Ex Vivo* Model of Retinal Degeneration via Antioxidant Activity and Targeting the SUMOylation

**DOI:** 10.1155/2022/8923615

**Published:** 2022-07-29

**Authors:** Kambiz Hassanzadeh, Zakaria Vahabzadeh, Lucia Buccarello, Jessica Dragotto, Massimo Corbo, Rita Maccarone, Marco Feligioni

**Affiliations:** ^1^Department of Biotechnological and Applied Clinical Sciences, University of L'Aquila, L'Aquila, Italy; ^2^Laboratory of Neuronal Cell Signaling, EBRI Rita Levi-Montalcini Foundation, Rome 00161, Italy; ^3^Cellular and Molecular Research Center, Research Institute for Health Development, Kurdistan University of Medical Sciences, Sanandaj, Iran; ^4^Need Institute, Milan, Italy; ^5^Department of Neurorehabilitation Sciences, Casa di Cura del Policlinico, Milan 20144, Italy

## Abstract

Retinal degeneration is the major and principal cause behind many incurable blindness diseases. Several studies indicated the neuroprotective effect of Curcuma longa in eye pathologies, specifically retinopathy. However, the molecular mechanism behind its effect has not been completely elucidated. Using an *ex vivo* model of retinal degeneration obtained from an *ex vivo* optic nerve cut (ONC), we demonstrated that Curcuma extract (Cur) exerted a neuroprotective effect. Importantly, Cur was able to modulate apoptosis and MAPK signaling pathway activation and prevent retinal ganglion cell (RGC) loss. Other well-known neuroprotective pharmacological tools, including memantine (Mem), citicoline (Cit), and ginkgolic acid (GA), were used to compare the potential mechanisms of Cur. The antioxidant activity of retinas treated with Cur following optic nerve cut was significantly higher than control, but Cur failed to change the retina glutamate content. Considering the antioxidant effect of Cur and taking advantage of our recent findings on the crosstalk between oxidative stress and post-translational protein modifiers, in particular, small ubiquitin-related modifier (SUMO), we were interested in exploring the effect of Cur on SUMOylation. We found that Cur significantly prevented the increase of protein SUMOylation, confirming our previous *in vitro* data indicating the cytoprotective effect of curcumin through modulating the oxidative stress and SUMO-JNK axis. Altogether, these results suggest that Curcuma protects the retina from degeneration via antioxidant activity and targets SUMOylation. Therefore, it might be considered for the combination therapy with other neuroprotective agents with different mechanisms in preclinical studies on retinal degeneration.

## 1. Introduction

Retinal degenerations, a group of heterogeneous disorders, are identified in different pathological conditions like age-related macular degeneration, glaucoma, or retinitis pigmentosa, presenting similar pathological features associated with retinal tissue damage [1–3]. Several molecular mechanisms have been reported to be involved in retinal degeneration and retinal ganglion cell (RGC) death in different eye pathologies. The excess of excitatory neurotransmitter glutamate release is, for instance, a key player in glaucoma retinal neurodegeneration, which initiates the activation of the N-methyl-D-aspartate (NMDA) receptor signaling cascade, which leads to an aberrant calcium intake within the cells, and impairs the mitochondrial function [4]. Furthermore, it is believed that the excitotoxicity-induced RGC death is caspase-dependent, being caspase inhibitors able to protect RGCs [5].

Oxidative stress, protein misfolding, deprivation of neurotrophins, and glial cell-induced inflammation in the retina are other essential players in retina cell death [3, 6, 7]. The retina is one of the highest oxygen-consuming tissues in the human body, making it highly susceptible to oxidative stress damage induced by reactive oxygen species (ROS) [8]. Oxidative stress, through increasing ROS, causes an imbalance between the prooxidant/antioxidant system and eventually leads to retina dysfunction and impairment in retinal pigment epithelium (RPE), endothelial cells, and retinal ganglion cells (RGCs) [9].

In addition, oxidative stress may interfere with dynamic processes in the cells, such as posttranslational modifications. Recently, we showed that oxidative stress induced by H_2_O_2_ increased protein SUMOylation in cells and activated the SUMO-1-JNK-Tau axis [10]. SUMOylation is a posttranslational modification that conjugates a small ubiquitin-like modifier (SUMO) peptide to the target protein. Dysregulation of SUMOylation has been indicated to be critically involved in several age-related disorders [11–13]. In this area, Sun et al. reported that SUMOylation critically manages retina and retinal pigment epithelial (RPE) aging, and targeting the SUMOylation pathway might be considered a potential therapeutic approach in the treatment of age-related macular degeneration [14].

Different methods are mimicking retina degeneration and RGC death. Recently, in our lab, we optimized an *ex vivo* model of retinal degeneration by cutting the optic nerve (ONC) that could replicate the retinal degeneration and represents a valuable model for primary screening of different compounds against retinal degeneration and RGC death [15].

Accumulating evidence during the past two decades provides a great deal of support to molecules able to perform neuroprotection through different molecular mechanisms. Several treatments are under investigation for retinal degeneration. However, the results are limited to specific pathologies and are not still satisfactory. The study of preclinical and clinical investigations on eye diseases demonstrates that curcumin has the potential to be used as a therapeutic agent in a variety of eye disorders such as glaucoma, diabetic retinopathy, cataract, age-related macular degeneration, conjunctivitis, pterygium, anterior uveitis, corneal neovascularization, and dry eye disease [16].

Curcumin is a natural compound found in the spice turmeric, the root of a plant that is scientifically known as Curcuma longa. Turmeric extract contains three curcuminoids, including curcumin (≈80%), demethoxycurcumin (≈15%), and bisdemethoxycurcumin (≈5%) [17]. Different forms and formulations of curcumin or curcuminoids have been tried for several purposes, and the obtained effects differ depending on the composition and pharmacokinetic parameters of the formulation [18]. Previously, we demonstrated that curcumin was able to prevent oxidative stress induced by H_2_O_2_, reduce cell death, and prevent SUMO-1 and c-Jun-N-terminal kinase (JNK) hyperactivation in SH-SY5Y cells [10].

Later, we found that cutting the optic nerve induces retinal degeneration in a time-dependent manner, and curcumin was able to prevent these pathological processes [15]. In accordance with these results, Ke et al. showed that pretreatment with curcumin significantly augmented the cell viability of BV-2 microglia in an *in vivo* rat model of chronic high intraocular pressure. They suggested that the antioxidant activity inhibits the oxidative damage of microglia which might be the mechanism of action for curcumin [19]. In another study, low doses of curcumin prevented staurosporine-induced ganglion cell death in both *in vitro* and *in vivo* studies. They showed that curcumin exerts its antiapoptotic effect through activation of NF-*κ*B, counteracting the staurosporine-mediated death of RGCs [20]. This antioxidant activity of curcumin has been considered mediated through its effects on the body's antioxidant defense system, free radicals scavenging, and/or by preventing lipid peroxidation [21].

Despite the different proposed mechanisms for the neuroprotective effect of curcuminoids, the exact mechanism of action has not been completely understood. Therefore, this study was aimed at evaluating the effect of Curcuma extract, which contains not only curcumin but a mix of curcuminoids and other compounds, on retinal degeneration in an *ex vivo* animal model of ONC. The possible activity of Curcuma extract on cell death and relevant mechanisms were evaluated by comparing these effects with pharmacological tools to understand more about the mechanism of action.

## 2. Materials and Methods

### 2.1. Cell Line Viability and Cytotoxicity Assay

The human neuroblastoma cell line, SH-SY5Y, was grown in DMEM medium (Thermo Fisher-GIBCO, 41966029) boosted with 1% L-glutamine (Thermo Fisher-GIBCO, 25030081), 10% FBS (Gibco, Invitrogen, USA), and 1% penicillin and streptomycin in a humidified incubator at 37°C with 5% CO2. The culture media was replaced every two to three days, and upon reaching 80% confluence, cells were split using 0.05% trypsin-EDTA. The cells were treated with different concentrations of Curcuma extract (1, 2.5, 5, 10, and 20 *μ*M). Curcuma extract was gifted by Linnea SA (Linnea, cat# CUM 1259), which has run a series of analyses showing that this, yellow powder of curcuma of Indian origin, contains 94.3% of total curcuminoids (HPLC assay). It is insoluble in water; the water content (KF) is 0.3% with sulfated ash of 1.1%. It has a residual solvent (GC) content of 1257 ppm. The powder does not contain pathogens, bacteria, or heavy metals. Cell viability was measured by incubating the cells with MTT (3-[4,5-dimethylthiazol-2-yl]-2,5-diphenyltetrazolium bromide). Briefly, in a 96-well plate, 20 *μ*l of MTT solution (4 mg/ml in PBS, 1X) was added to 25.000 cells and incubated for 4 hours at 37°C. Subsequently, the formazan product produced by the viable cells was dissolved in 1 : 25 HCl 1 N with isopropanol, and the optical density was read at 540 nm using an automated spectrophotometer. In addition, lactate dehydrogenase (LDH) assay was performed using the cytotoxicity assay kit (CyQUANT™ LDH Cytotoxicity Assay nr #C20300 Thermo Fisher) according to the manufacturer protocol. The cell medium was collected, and the lactate dehydrogenase (LDH) released in the medium was quantified to obtain the toxicity induced by the Curcuma extract.

### 2.2. Animals and Treatment

C57BL/6J mice (male, 20–30 g, 8-10 weeks; Jackson Laboratories, USA) were rendered and housed (4 animals per group) in a temperature- and humidity-controlled condition (12 h dark/light cycle, lights on 07 : 00 a.m.) with free access to food and water. Animals were euthanized in a CO_2_ chamber (CO_2_ plus 10–50% O_2_). The study was performed according to the guidelines of the national and international laws and policies (EU Directive 2010/63/EU for animal experiments) and approved by the Ethical Committee on animal experiments of EBRI “Rita Levi-Montalcini” Foundation (Rome, Italy). The scientific project was approved by the Italian Ministry of Health (Permit Number F8BBD.N.7OK).

The total number of animals used in this study was 60. Animals were sacrificed, and their eyes were harvested according to our *ex vivo* model of retinal degeneration induction [15]. The enucleated eyeballs were used freshly (WT) or injected with different treatments: Ctr: dimethyl sulfoxide (DMSO, 0.05%), or Curcuma extracts: Cur 5 or 10 *μ*M (Linnea, cat# CUM 1259), or memantine: Mem 10 *μ*M (ChemCruz, cat# SC-203628), or citicoline: Cit 100 *μ*M (Santa Cruz, cat# SC-300380), or ginkgolic acid: GA 20 *μ*M (Calbiochem, cat# 345887).

The treated eyes were kept in PBS at 4°C, and after 24 hours, retina was dissected and processed for staining, immunofluorescence, or biochemical analysis (*n* = 5 for each experimental group).

Cur and GA first were dissolved in DMSO and then diluted in PBS to a final concentration of 5 or 10 *μ*M. Other treatments were dissolved in PBS. The above-mentioned concentrations of drugs used in this study were adapted from previous investigations indicating the neuroprotection of memantine and citicoline [22, 23] and SUMO inhibitory activity of ginkgolic acid [24]. The final volume of injection inside the eye was adjusted to 1 *μ*l, and a randomization procedure was performed for allocation concealment of animals' eyes to vehicle(s) or treatment(s).

### 2.3. Preparation of Retinal Lysates

After enucleating the eyes, the retinas immediately were extracted and lysated in 100 *μ*l of a lysis buffer solution containing (mM): TRIS acetate, 20; sucrose, 0.27; EDTA, 1; EGTA, 1; Na orthovanadate, 1; NaF, 50; Na pyrophosphate, 5; Na *β*-glycerophosphate, 10; and DTT, 1 (Sigma-Aldrich, Milan, Italy); 1% Triton X-100 (Sigma-Aldrich, Milan, Italy, nr 9002-93-1); a complete set of protease inhibitors (Complete, Roche Diagnostics, Basel, Switzerland) and phosphatase inhibitors (Sigma, St. Louis, MO); and N-ethylmaleimide (NEM, Sigma-Aldrich, Milan, Italy, nr 128-53-0). Samples were then sonicated, and the homogenates were placed on ice for 30 min to allow protein solubilization. Then, they were centrifuged at 10000 rpm for 10 min, and subsequently, the supernatant was collected and stored at −80°C until needed.

### 2.4. Western Blot

The Bradford Assay (Bio-Rad Protein Assay 500-0006, Munchen, Germany) was used to quantify the protein concentration of samples. Fifty micrograms of extracted proteins were separated on 12% SDS polyacrylamide gel electrophoresis (SDS-PAGE) and transferred to PVDF membranes. Then, the membranes were blocked in Tris-buffered saline (5% nonfat milk powder, 0.1% Tween 20) for one hour at room temperature. They were incubated overnight at 4°C with the following primary antibodies diluted in the same buffer: anti-*β*-actin (Abcam, Cambridge, UK. Cat#ab8227), anti-BAX (Cell Signaling, Danvers, MA, USA. cat#5023), anti-BCL2 (Cell Signaling, Danvers, MA, USA. cat#15071), anti-BCL-XL (Cell Signaling, Danvers, MA, USA. cat#2764), anti-BRN3a (Santa Cruz Biotechnology, Milan, Italy Cat#C-20 sc31984), anti-cleaved caspase 3 (Cell Signaling, Danvers, MA, USA. cat #9654), anti-p-c-Jun (Cell Signaling, Danvers, MA, USA. cat#9164), anti-c-Jun (Cell Signaling, Danvers, MA, USA. cat#9165), anti-p-ERK (Cell Signaling, Danvers, MA, USA. cat#4377), anti-ERK (Cell Signaling, Danvers, MA, USA cat#4695), anti-LC3B (Cell Signaling, Danvers, MA, USA. cat#43566), anti-p53 (Cell Signaling, Danvers, MA, USA. cat#2527), anti-p-JNK (Cell Signaling, Danvers, MA, USA. cat#9251), anti-JNK (Cell Signaling, Danvers, MA, USA. cat#9252), anti-NeuN (Cell Signaling, Danvers, MA, USA. cat. #24307), anti-SUMO-1 (Santa Cruz Biotechnology, Milan, Italy Cat#D-11sc-5308), anti-SUMO-2,3 (Cell Signaling, Danvers, MA, USA. cat# 4971), anti-UBC9 (Cell Signaling, Danvers, MA, USA. cat. #4786), anti-ubiquitin (Cell Signaling, Danvers, MA, USA. cat#3933). To develop the blots, horseradish peroxidase-conjugated secondary antibodies (anti-mouse or anti-rabbit, 1 : 5000, Santa Cruz Biotechnology, Milan, Italy) were utilized, and the immunoreactive bands were visualized by exposure to the ECL chemiluminescence system (Cyanagen, westar antares nr XLS142, Bologna, Italy). Beta-actin was used as the loading control for quantification. The ratio of phosphorylated form to the total protein was calculated to quantify JNK, c-Jun, and ERK activation. The ratio between LC3BII and LC3BI isoforms was measured to detect the conversion as a biomarker to detect autophagy. Western blots were quantified by densitometry using ImageJ software.

### 2.5. Total Antioxidant Capacity (TAC) Assay

The antioxidant efficiency of the treatments was measured using the total antioxidant capacity kit (Abcam, Cambridge, UK, Cat#ab65329) according to the manufacturer's instructions. Briefly, the retinas were washed in cold PBS, and 10 mg of the retina was resuspended in 500 *μ*l of ice-cold PBS. Tissues were homogenized with a Dounce homogenizer, incubated for 10 minutes on ice, and centrifuged for 5 minutes at 4°C at top speed to remove any insoluble material. The supernatant was collected and transferred to a new tube. It was allowed to reduce Cu^2+^ for 90 minutes at room temperature, and the output was measured on a microplate reader at OD 570 nm. A Trolox standard curve was plotted (supplementary figure 1), and the results were expressed as Trolox equivalent according to the standard curve [25].

### 2.6. Histological Staining and Immunofluorescence Analysis

The enucleated eyes were fixed in cold methanol: acetic acid: PBS (3 : 1 : 4) overnight [15]. Then, they were incubated in 30% sucrose overnight, and finally, they were embedded in an optimal cutting temperature (OCT, Sigma, St. Louis, MO, USA) compound. Eyes were cut at a thickness of 12 *μ*m. For reliability, the sections containing the optic nerve were utilized, and in each eye, at least five discontinuous sections were analyzed. Thickness was measured starting at the optic nerve head and extending along the vertical meridian to the superior and inferior ora serrata. Retinal cryosections were mounted and stained with hematoxylin-eosin (H&E) (Sigma-Aldrich), coverslipped with Eukitt, and observed under a light-transmission microscope (Nikon) [26].

For immunofluorescence analysis, retinal cryosections were first permeabilized with PBS-Triton X-100 (Fluka) 0.5% for 15 minutes, then washed three times with PBS, and incubated in a blocking solution (glycine 0.1 M in PBS, FBS 2%, BSA 2%) for 1 hour in a humid chamber. The primary antibody anti-SUMO-1 (1 : 100, Santa Cruz Biotechnology, Milan, Italy, Cat#D-11sc-5308) was added and incubated overnight at 4°C. The next day, samples were incubated in Alexa-568 (Red) (1 : 500; Invitrogen Thermo Fisher, Milan, Italy) diluted in PBS for 45 minutes in a humid chamber. DAPI (1 : 500; Invitrogen Thermo Fisher, Milan, Italy) was used for nuclear staining, and finally, coverslips were mounted in Fluorsave mounting medium (Calbiochem, Millipore, Billerica, MA, USA, nr 345789) [27]. Images were acquired with an Olympus microscope equipped with an Olympus confocal scan unit (microscope BX61 and Confocal system FV500) managed by AnalySIS Fluoview software with 3 laser lines used to detect DAPI staining and secondary antibodies. The magnifications of the images were 20×. The software controls and microscope settings such as scan speed, pinhole aperture, optical zoom, and image resolution were kept uniform. Confocal images were processed with ImageJ software.

### 2.7. Determination of Glutamate in the Retina by High-Performance Liquid Chromatography (HPLC)

O-Phthalaldehyde (OPA, Sigma-Aldrich, Germany), 2-mercaptoethanol (2-ME, Sigma–Aldrich, Germany), glutamate (analytical grade external standard, Sigma-Aldrich, Germany), homoserine (analytical grade internal standard, Sigma-Aldrich, Germany), potassium tetraborate (Sigma-Aldrich, Germany), HPLC grade methanol (Merck), and ultrapure water were used for this experiment.

A calibration curve was prepared using an injection of the freshly provided glutamate standard solutions at different concentrations including 0.05, 0.1, 0.5, 5, 10, and 20 *μ*M (Supplementary figure 2). To prepare the samples for injection into the HPLC system, each retina sample was homogenized in 250 *μ*l of acetate buffer (0.05 M)/methanol (790 ml: 210 ml) containing homoserine as an internal standard at a final concentration of 6 *μ*M. The samples were then sonicated (using 50% power, 0.5 cycles, three times totally for 30 seconds) and centrifuged at 15,000*g* for 10 min at 4°C. The supernatant (or each of the standard solutions) was transferred to a microtube containing a mixture of 100 *μ*l borate buffer (pH 9.9) and 50 *μ*l freshly prepared methanolic solution of OPA (10 mg/ml). After 110 seconds, 25 *μ*l of HCL 0.7 M was added and vortexed for 5 sec. 250 *μ*l of acetate buffer (0.05 M)/Methanol (790 ml: 210 ml) was then added immediately and vortexed. Finally, 100 *μ*l of each cleared sample or standard was injected into the HPLC system [28].

The HPLC analysis was performed using KNAUER HPLC (KNAUER GmbH, Germany). An HPLC column, Hypersil™ ODS C18 Column 5 *μ*, 4.6 × 100 mm (KNAUER GmbH, Germany) at column temperature 35°C, was applied for chromatographic separation using a mobile phase containing a mixture of solvent A (acetate buffer (0.05 M)/methanol (790 ml: 210 ml) and solvent B (acetate buffer (0.05 M)/methanol (250 ml: 750 ml) in the gradient mode and flow rate 1 ml/min for 30 min. The detection was applied at Ex: 340 nm and Em: 450 nm using RF-10AXL fluorescence detector (KNAUER GmbH, Germany).

### 2.8. Data Analysis

All data are expressed as mean ± SEM for five independent experiments, and a *p* value of < 0.05 was considered statistically significant. Statistical analysis was performed using SPSS (version 19). All quantifications were analyzed using one-way ANOVA, followed by Tukey's post hoc test.

## 3. Results

### 3.1. Effect of Curcuma Extract on Apoptotic Pathway Activation in the Retina following Optic Nerve Cut

**Figure 1:**
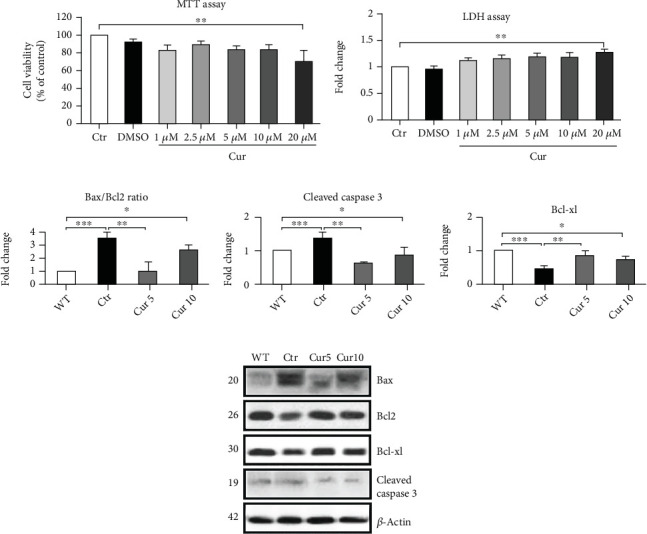
Effect of Curcuma extract on the cell viability assay and apoptotic signaling markers. (a) MTT assay has been done to check the cell viability in the presence of different concentrations of Curcuma extract. (b) LDH assay was performed to evaluate the cell death. (c–f) Representative western blots and relative quantifications in different treated groups. Data are shown as mean ± SEM. One-way ANOVA followed by Tukey's post hoc test was used to analyze the statistical differences among groups. A *p* value of less than 0.05 was considered significant in all analyses. ⁣^∗^*p* < 0.05, ⁣^∗∗^*p* < 0.01, and ⁣^∗∗∗^*p* < 0.001. WT: nontreated fresh retina; Ctr: control; Cur: Curcuma extract.

To test the toxic profile of Curcuma extract and to choose a safe concentration to be used in our experiments, we used increasing concentrations of Curcuma extract on a neuronal-like cell culture, SH-SY5Y cells, investigating the cell viability using MTT assay and cell cytotoxicity as a release of lactate dehydrogenase (LDH). The results showed a significant decrease in cell viability in the group treated with Curcuma extract at 20 *μ*M compared to the control (Ctr: 100 vs. Cur 20 *μ*M: 71.97 ± 10.32, *p* < 0.01; Figure 1(a)). In addition, the same concentration induced a significant increase in LDH release compared to the control (Ctr: 1 vs. Cur 20 *μ*M: 1.25 ± 0.02, *p* < 0.01; Figure 1(b)). These results induced us to use Cur at 5 (Cur 5) and 10 *μ*M (Cur 10) in the *ex vivo* mouse model of ONC [15].

First, we checked the involvement of the apoptotic pathway by treating the eyes with DMSO (0.05%) or Cur at 5 or 10 *μ*M, and we found a significant increase of proapoptotic markers in the control group compared to the untreated eyes (WT): Bax/Bcl2 ratio (Ctr: 3.54 ± 0.4 vs. WT:1, *p* < 0.001; Figure 1(c)) and cleaved caspase 3 (Ctr: 1.34 ± 0.1 vs. WT:1, *p* < 0.05; Figure 1(d)). Only Cur 5 was able to prevent significantly the augmentation of Bax/Bcl2 ratio (Ctr: 3.54 ± 0.4 vs. Cur 5: 0.97 ± 0.76, *p* < 0.01; Figure 1(c)) and caspase3 (Ctr: 1.34 ± 0.1 vs. Cur 5: 0.63 ± 0.04, *p* < 0.05; Figure 1(d)). In addition, Bcl-xl, the anti-apoptotic marker, significantly decreased in the control group compared to the WT (Ctr: 0.45 ± 0.12 vs. WT: 1, *p* < 0.01; Figure 1(e)) and Cur 5 prevented fall significantly (Ctr: 0.45 ± 0.12 vs. Cur 5: 0.83 ± 0.1, *p* < 0.05; Figure 1(e)).

### 3.2. Effect of Curcuma Extract on Mitogen-Activated Protein Kinase (MAPK) Pathway

**Figure 2:**
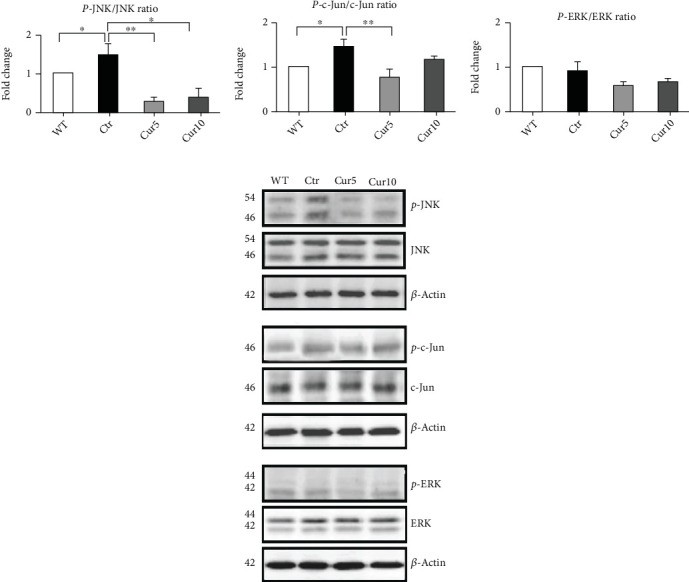
Effect of Curcuma extract on mitogen-activated protein kinase (MAPK) pathways. Representative western blots and relative quantifications showed a significant change in (a) p-JNK/JNK and (b) p-c-Jun/c-Jun but not (c) p-ERK/ERK in retina homogenates collected from different treatments. Data are shown as mean ± SEM. One-way ANOVA followed by Tukey's post hoc test was used to analyze the statistical differences among groups. A *p* value of less than 0.05 was considered significant in all analyses. ⁣^∗^*p* < 0.05 and ⁣^∗∗^*p* < 0.01. WT: nontreated fresh retina; Ctr: control; Cur: Curcuma extract.

After demonstrating the antiapoptotic effect of Curcuma in our *ex vivo* model, we were interested in checking its impact on the activation of the most critical players in MAPK signaling: c-Jun N-terminal kinases (JNKs), c-Jun (c-Jun), and extracellular signal-regulated kinases (ERKs). Our findings revealed activation of JNK in control compared to the WT group (Ctr: 1.48 ± 0.27 vs. WT: 1, *p* < 0.05; Figure 2(a)); in addition, the phosphorylated form of c-Jun as the JNK substrate showed an increase in the control group significantly (Ctr: 1.44 ± 0.13 vs. WT: 1, *p* < 0.05; Figure 2(b)). Analyzing the data related to the ERK activation showed that there was not a statistical difference among groups (Figure 2(c)). Intriguingly, we found that Curcuma extract in both doses used in this study was able to prevent the JNK activation (Ctr: 1.48 ± 0.27 vs. Cur 5: 0.26 ± 0.1, *p* < 0.01, and vs. Cur 10: 0.39 ± 0.22, *p* < 0.05; Figure 2(a)). Moreover, Cur 5 could significantly prevent the phosphorylation of c-Jun in a pattern similar to that of JNK (Ctr: 1.44 ± 0.13 vs. Cur 5: 0.77 ± 0.15, *p* < 0.01; Figure 2(b)).

### 3.3. Curcuma Extract Protected Retinal Damage in an *Ex Vivo* Model of ONC

**Figure 3:**
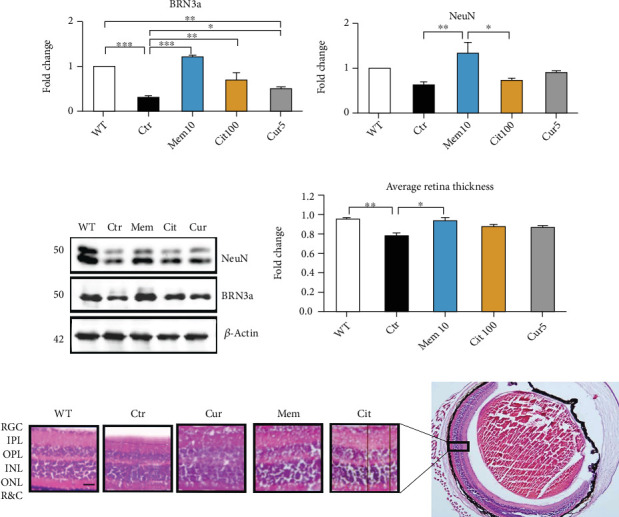
Curcuma extract protected retinal damage in an *ex vivo* model of toxicity induction. (a–c) Representative western blots and relative quantifications for NeuN as the neuronal marker and BRN3a, a specific marker for RGCs, in retina homogenates collected from different treatments. (d and e) Representative histological (hematoxylin and eosin) staining and relative quantifications for retina thickness assay. Scale bar = 50 *μ*m. Data are shown as mean ± SEM and were normalized to the nontreated group (WT). One-way ANOVA followed by Tukey's post hoc test was used to analyze the statistical differences among groups. A *p* value of less than 0.05 was considered significant in all analyses. ⁣^∗^*p* < 0.05, ⁣^∗∗^*p* < 0.01, and ⁣^∗∗∗^*p* < 0.001. WT: nontreated fresh retina; Ctr: control; Cur: Curcuma extract; Mem: memantine; Cit: citicoline.

Continuing our experiments, we decided to use Cur 5 as the more potent dose in this study. In an *ex vivo* model of ONC, the eyeballs were enucleated and kept in PBS at 4°C showed retinal degeneration in 24 h [15]. DMSO injected into the enucleated mouse eyeballs as the control group induced BRN3a as a specific marker for retinal ganglion cells, a significant fall compared to the WT group (Ctr: 0.29 ± 0.06 vs. WT: 1, *p* < 0.001; Figure 3(a)). NeuN, the most widely used neuronal marker in neuroscience research, also tended to decrease in control; however, it was not statistically significant compared to the WT (Figure 3(b)). Hematoxylin-eosin staining of the retina showed a decrease in the retinal layers' thickness in the control group compared to the WT (Ctr: 0.8 ± 0.03 vs. WT: 1, *p* < 0.05; Figure 3(d)), showing the damage to the retina 24 h after optic nerve cut. To clarify the mechanism of action and to better understand the possible effect of Curcuma, we compared the effect of Cur with two well-known neuroprotective pharmacological tools with different mechanisms, memantine and citicoline. Our findings revealed that memantine was able to prevent the fall in BRN3a (Ctr: 0.29 ± 0.06 vs. Mem: 1.2 ± 0.04, *p* < 0.001; Figure 3(a)) and NeuN (Ctr: 0.66 ± 0.07 vs. Mem: 1.4 ± 0.26, *p* < 0.01; Figure 3(b)) significantly. Moreover, according to the western blot results, we found that Cit and Cur were able to rescue the BRN3a (Ctr: 0.29 ± 0.06 vs. Cit: 0.7 ± 0.15, *p* < 0.01, and vs. Cur 5: 0.52 ± 0.02, *p* < 0.05; Figure 3(a)) significantly but not NeuN. There was a similar pattern for these effects on retinal layers' thickness. Although all treatments tended to prevent the decrease in retinal thickness, just Mem significantly prevented the average retina thickness (Ctr: 0.8 ± 0.03 vs. Mem: 0.98 ± 0.44, *p* < 0.05; Figure 3(d)).

### 3.4. Effect of Curcuma on the Apoptotic Pathway Was Comparable to Memantine and Citicoline

**Figure 4:**
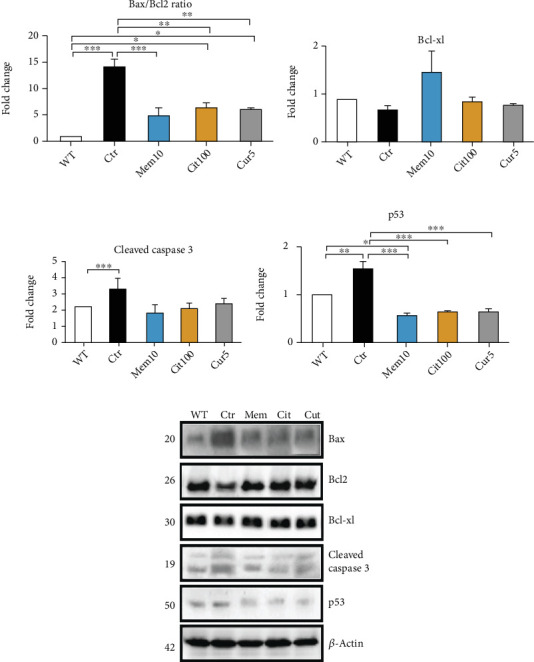
Comparison of the effect of Curcuma extract with memantine and citicoline on apoptotic markers. Representative western blots and relative quantifications for apoptotic markers, including (a) Bax/Bcl2 ratio, (b) Bcl-xl, (c) caspase3, and (d) p53 in retina homogenates collected from different treatments. Data are shown as mean ± SEM and were normalized to the nontreated group in western blot (WT). One-way ANOVA followed by Tukey's post hoc test was used to analyze the statistical differences among groups. A *p* value of less than 0.05 was considered significant in all analyses. ⁣^∗^*p* < 0.05, ⁣^∗∗^*p* < 0.01, and ⁣^∗∗∗^*p* < 0.001. WT: nontreated fresh retina; Ctr: control; Cur: Curcuma extract; Mem: memantine; Cit: citicoline.

Comparing the antiapoptotic effect of Cur with Mem and Cit, we found that all treatments could prevent the dramatic increase of Bax/Bcl2 ratio induced by optic nerve cut significantly (Ctr: 14.08 ± 1.03 vs. Mem: 4.83 ± 1.55, *p* < 0.001; vs. Cit: 6.28 ± 0.89, *p* < 0.01; and vs. Cur: 6.2 ± 0.11, *p* < 0.01; Figure 4(a)). In a similar pattern, all treatments were able to prevent the p53 augmentation occurred in the control group (Ctr: 1.55 ± 0.17 vs. Mem: 0.53 ± 0.06, *p* < 0.001; vs. Cit: 0.61 ± 0.006, *p* < 0.001; and vs. Cur: 0.64 ± 0.08, *p* < 0.001; Figure 4(d)). Cleaved caspase 3 and Bcl-xl showed a tendency to decrease and increase in treated eyes, respectively; however, they were not statistically significant (Figures 4(b) and 4(c)).

### 3.5. Total Antioxidant Capacity and Retina Glutamate Content in Different Treated Groups

**Figure 5:**
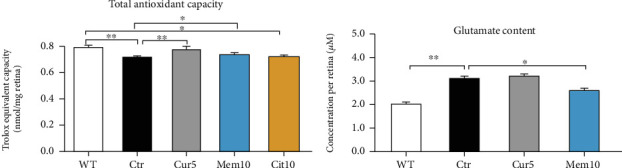
Total antioxidant capacity assay and retina glutamate content in different groups. (a) Trolox equivalent capacity measured in mouse retina lysates, showing quantity (nmol) per mg of retina tissue. (b) Comparison of the retina glutamate content between Curcuma extract treatment and memantine using HPLC. Data are shown as mean ± SEM. One-way ANOVA followed by Tukey's post hoc test was used to analyze the statistical differences among groups. A *p* value of less than 0.05 was considered significant in all analyses. ⁣^∗^*p* < 0.05 and ⁣^∗∗^*p* < 0.01. WT: nontreated fresh retina; Ctr: control; Cur: Curcuma extract; Mem: memantine; Cit: citicoline.

Comparing the total antioxidant capacity (TAC) in different groups, we found that it was significantly decreased in the control retina compared to the nontreated group (WT) (WT: 0.76 ± 0.006 vs. Ctr: 0.68 ± 0.009 nmol Trolox eq/mg, *p* < 0.01). Cur 5 and memantine, but not citicoline, significantly prevented the decline in the TAC (Ctr: 0.68 ± 0.009 vs. Cur 5: 0.75 ± 0.009 nmol Trolox eq/mg, *p* < 0.01, and vs. Mem: 0.71 ± 0.007 nmol Trolox eq/mg, *p* < 0.05) (Figure 5(a)).

Although glutamate plays an essential role in the physiological function of the retina as an excitatory neurotransmitter, excessive glutamate can be toxic to retinal neurons through overstimulation of its receptors and causes apoptotic neuronal cell death at high concentrations. Since the protective effect of memantine is mostly related to the blockage of glutamate release [29], we were curious to explore whether this mechanism was involved in the protective and antiapoptotic effects of memantine and Curcuma. We found that there was a significant increase in glutamate content in the control group that received DMSO compared to the WT (Ctr: 3.13 *μ*M ± 0.14 vs. WT: 2.08 ± 0.17, *p* < 0.01) and that memantine was able to prevent the augmentation of retina glutamate content significantly (Ctr: 3.13 *μ*M ± 0.14 vs. Mem: 2.6 ± 0.17, *p* < 0.05), while Cur treatment was not effective to contrast the retina glutamate elevation (Figure 5(b)).

### 3.6. Curcuma Extract Affects the SUMOylation and Ubiquitination in the Retinal Degeneration Model

Recently, we found that curcumin influenced the retina SUMO-1ylation following the optic nerve cut [15]. Here, we explored Cur effect of SUMO-1 and SUMO-2,3ylation by biochemistry and immunofluorescence assays.

**Figure 6:**
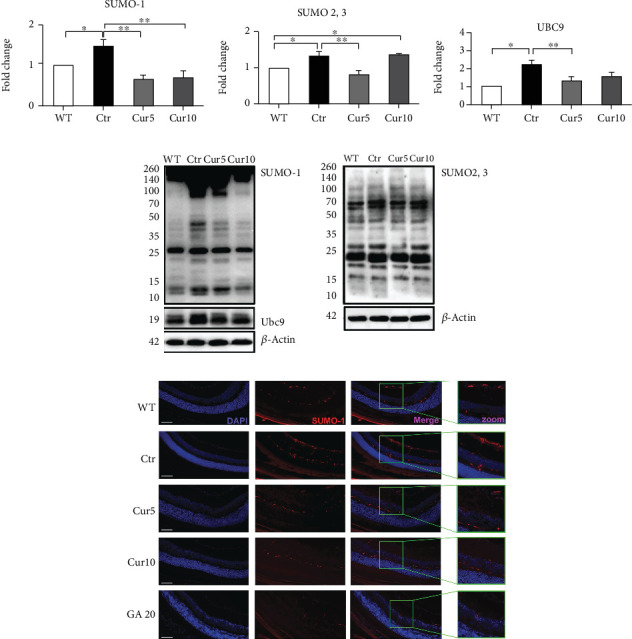
Effect of Curcuma extract on SUMOylation in the *ex vivo* model of retinal degeneration. Representative western blots and relative quantifications for (a) SUMO-1, (b) SUMO-2,3, and (c and d) Ubc9 in retina homogenates collected from different treatments. (e) Immunofluorescence analysis of retinal sections collected from different treatments. IF images indicated an increase of SUMO-1-positive cells (red dots) in samples collected from the Ctr, and Cur 5 and Cur 10 were able to prevent the SUMO-1 positive cell augmentation. Nuclei were stained with the nuclear marker DAPI (blue). Scale bar = 200 *μ*m. In the western blot analysis, data are shown as mean ± SEM and were normalized to the nontreated group (WT). One-way ANOVA followed by Tukey's post hoc test was used to analyze the statistical differences among groups. A *p* value of less than 0.05 was considered significant in all analyses. ⁣^∗^*p* < 0.05 and ⁣^∗∗^*p* < 0.01. WT: nontreated fresh retina; Ctr: control; Cur: Curcuma extract; GA: ginkgolic acid.

In the *ex vivo* ONC, there were significant increases in both SUMO-1 (Ctr: 1.46 ± 0.18 vs. WT: 1, *p* < 0.05; Figure 6(a)) and SUMO-2,3ylation (Ctr: 1.33 ± 0.08 vs WT: 1, *p* < 0.05; Figure 6(b)) level in the control group compared to the WT. Both doses of Cur used in this study prevented the induction of SUMO-1ylation compared to the control (Ctr: 1.2 ± 0.06 vs. Cur 5: 0.64 ± 0.09, *p* < 0.01, and vs. Cur 10: 0.7 ± 0.12, *p* < 0.01; Figure 6(a)); however, only Cur 5 was able to inhibit SUMO-2,3 augmentation significantly (Ctr: 1.33 ± 0.08 vs. Cur 5: 0.8 ± 0.12, *p* < 0.01; Figure 6(b)). To compare the effect of Curcuma extract with a standard SUMOylation inhibitor, ginkgolic acid [30] was injected into the eyes as a positive control group. SUMO-1 immunofluorescence staining results confirmed the biochemical assays showing a more immunoreactive SUMO-1ylation in the retina in the control group that appeared less present after Curcuma extract treatment which was similar to the GA group (Figure 6(e)). Moreover, we investigated the expression level of the SUMO E2 enzyme, Ubc9 [13]. Following optic nerve cut, there was a dramatic increase in Ubc9 in control in comparison with WT (Ctr: 2.23 ± 0.32 vs. WT: 1, *p* < 0.01; Figure 6(c)), while Cur 5 significantly inhibited Ubc9 augmentation compared to the control (Ctr: 2.23 ± 0.32 vs. Cur 5: 1.35 ± 0.23, *p* < 0.05; Figure 6(c)) and maintained its level close to the WT group.

**Figure 7:**
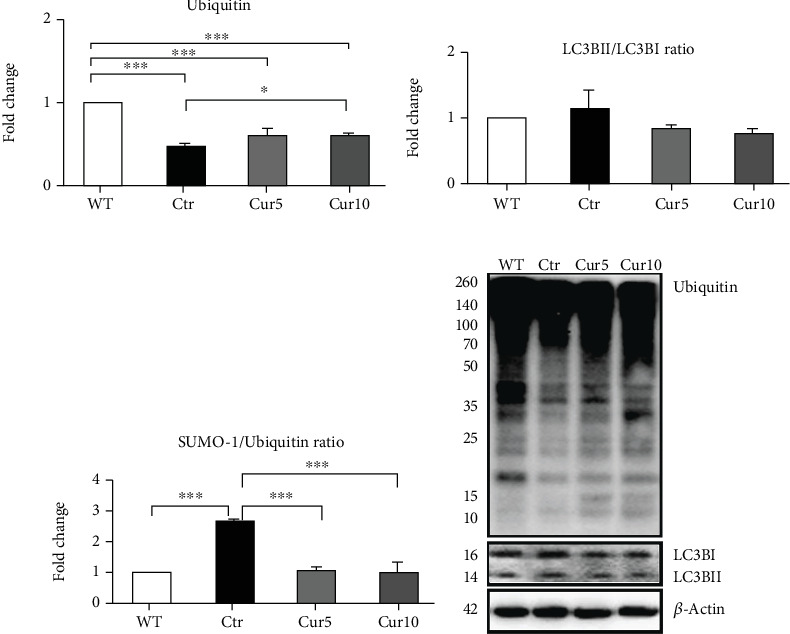
Effect of Curcuma extract on ubiquitination and autophagy marker in the *ex vivo* model of retinal degeneration. Representative western blots and relative quantifications for (a) ubiquitin and (b) LC3B, the autophagy marker, in retina homogenates collected from different treatments. (c) A SUMO-1/ubiquitin ratio quantification. Data are shown as mean ± SEM and were normalized to the nontreated group (WT). One-way ANOVA followed by Tukey's post hoc test was used to analyze the statistical differences among groups. A *p* value of less than 0.05 was considered significant in all analyses. ⁣^∗^*p* < 0.05 and ⁣^∗∗∗^*p* < 0.001. WT: nontreated fresh retina; Ctr: control; Cur: Curcuma extract.

Evaluating the UPS and autophagy degradation systems revealed that ubiquitin level was dramatically dropped in the control compared to the WT (Ctr: 0.47 ± 0.03 vs. WT: 1, *p* < 0.001; Figure 7(a)), but the LC3B conversion, the activation of LC3B-I to LC3B-II, was not significantly changed (Figure 7(b)). Moreover, we found that Curcuma extract at both doses was not able to rescue the ubiquitin level drop that it was maintained close to the control group (Ctr); however, in the Cur 10 treatment, the ubiquitin level was significantly higher than control (Ctr: 0.47 ± 0.03 vs. Cur 5: 0.62 ± 0.01, *p* < 0.05; Figure 7(a)). Analyzing the data obtained from western blot for autophagy marker, LC3B, we observed a tendency to decrease the LC3B-II/LC3B-I ratio in the groups treated with Curcuma extract, but the changes were not statistically significant (Figure 7(b)).

Taking advantage of the fact that there is a crosstalk and mutual regulation between SUMO and ubiquitin in physiological and pathological conditions [31], we calculated the SUMO-1/ubiquitin ratio to understand the effect of Curcuma treatment in the model of retinal degeneration. We found that the SUMO-1/ubiquitin ratio was significantly raised in control compared to the WT (Ctr: 2.6 ± 0.04 vs. WT: 1, *p* < 0.001; Figure 7(c)), and more intriguingly, Curcuma extract at both doses prevented these changes significantly (Ctr: 2.6 ± 0.04 vs. Cur 5: 1.04 ± 0.02, *p* < 0.001, and vs Cur 10: 1.02 ± 0.2, *p* < 0.001; Figure 7(c)) and maintained the ratio close to the WT.

## 4. Discussion

Recently, we found that curcumin was able to rescue the retina from the degeneration [15]. Although curcumin is the most important curcuminoid, 75% of the active ingredient, there are other curcuminoids in the Curcuma extract that might affect the pharmacokinetics and pharmacodynamics of this compound. Therefore, in this study, we investigated the effect of Curcuma extract, and we compared it with well-known pharmacological agents with different mechanisms of action, such as citicoline, memantine, and ginkgolic acid, to better understand the mechanisms behind the function of Curcuma in retinal protection.

Several mechanisms have been highlighted for the neuroprotective function of citicoline, such as mitochondrial protection, increased activity of glutathione synthesis, restoration of phosphatidylcholine levels, reduction of lipid peroxidation, and attenuation of free fatty acid release [32].

In the different paradigms of experimental glaucoma, citicoline exerted antiapoptotic effects on damaged RGCs by decreasing the activity of caspase 9 and caspase 3 [33]. Systemic administration of citicoline in an optic nerve crash protected RGCs and their axons from neurodegeneration and augmented retinal expression of the antiapoptotic protein Bcl-2 [34].

Another neuroprotective agent is memantine, an uncompetitive N-methyl-D-aspartate (NMDA) glutamate receptor antagonist, which has been demonstrated to be neuroprotective in glaucoma in different animal models [35, 36]. However, there has been less convincing evidence in human studies, and unfortunately, clinical trials failed to prove the protective effect of memantine in human glaucoma patients [37]. Different mechanisms of action have been proposed for the neuroprotective effect of memantine in the brain and retina pathologies; among them, its action on the glutamate and NMDA-receptor might be the most critical. Memantine rescues neurons by blocking the excessive glutamate-receptor activation, which contributes to the pathobiology of the neurodegeneration [29].

We recently optimized an *ex vivo* model of retinal degeneration. We confirmed that enucleation of the eyeball with optic nerve cut and injection of PBS inside the eye, kept for 24 h at 4°C in physiological solution, could complete the degeneration process. Taking advantage of this easy and noninvasive method, in this study, we injected the Curcuma extract or other pharmacological tools, including memantine, citicoline, or ginkgolic acid, into the eyes. First, we found that Curcuma was able to prevent cell death by exploring the apoptotic markers. Curcuma prevented the increase in proapoptotic markers, and the decrease in antiapoptotic marker induced in the *ex vivo* model of retina degeneration. We and others previously obtained similar findings [38–40].

Comparing the Curcuma with other neuroprotective agents, we found that the antiapoptotic function of Curcuma was comparable with memantine and citicoline. The results also revealed that p53, the protein that regulates the repair of cellular DNA and induces apoptosis, was increased in the control group, and therapies were able to prevent this augmentation in a similar pattern. The antiapoptotic effect of Curcuma through inhibition of p53 activation has been reported before [41, 42]. The pattern of p53 changes in the model of neurodegeneration and the effect of treatment is very similar to the Bax/Bcl2 ratio results, confirming the previous evidence that activation of p53 directly induces the transcription of Bax and inhibits Bcl-2 [43].

Exploring the MAPK activity in our samples, we found the protective effect of Curcuma through inhibiting the JNK activity. These results confirmed our and others' findings indicating the important role of JNK in oxidative stress and the mechanism of the antioxidant effect of curcumin through JNK inhibition [10, 15, 44]. Like other parameters explained before, there are also results reporting the JNK inducer function for curcumin [45].

To find the outcome of our treatment on the retina, the NeuN, and BRN3a, neuronal and RGC markers, respectively, were analyzed in the retina, and we found that Curcuma was able to rescue the retinal ganglion cells by preventing the fall of RGCs induced in our model of degeneration. Furthermore, citicoline provided the same pattern in this analysis, but memantine was more potent than the other treatments. It was able to protect not only the RGCs but also the neuronal marker, NeuN, significantly, indicating its more effectiveness in rescuing the retina. A similar result was obtained in the retina thickness assay in which we found that only memantine was able to maintain the retina thickness significantly, while Curcuma and citicoline failed to perform this function. These effects induced us to check the glutamate content of the retina and explore the effect of Curcuma compared to the memantine. It is noteworthy that like other parts of the central nervous system (CNS), RGCs are susceptible to glutamate-induced excitotoxicity [46].

Analyzing the data obtained from HPLC, we found a significant increase in retina glutamate content after the optic nerve cut. This was in line with the evidence suggesting that optic nerve injury activates retinal astrocytes and microglia, inducing the release of glutamate into the extracellular space [47]. Concerning the memantine mechanism of action, its function in preventing the retina glutamate content was expectable, while Curcuma was not able to prevent the glutamate amount showing that it probably protects the retina through other signaling mechanism.

Previously, we and others demonstrated the antioxidant activity of curcumin in different *in vitro* and *in vivo* studies utilizing cells or different organs and pathologies [10, 48, 49]. In this study, we found that Curcuma extract provides a significant antioxidant activity in the retina, which was in line with the results indicating that the antioxidant effect of curcumin plays a pivotal role in its neuroprotective effect [50, 51]. Curcuma extract also includes demethoxycurcumin and bisdemethoxycurcumin, which have been recognized to be natural antioxidants [52, 53]. Therefore, Curcuma's total antioxidant capacity might be higher than the pure curcumin we used in our previous study. The antioxidant property of Curcuma has also been reported and discussed in retinal epithelial cells. Kim et al. investigated the antioxidant protectant efficacy of curcumin in the forms of nanospheres. They demonstrated that the nanoformulated curcumin provided a significantly decreased ROS production in retinal epithelial cells [54].

The last mechanism explored for the Curcuma extract effect was SUMOylation and ubiquitination. Both biochemical and immunofluorescence assays showed that Curcuma decreased the SUMOylation, and SUMO level was decreased even more than the control. This effect was comparable to ginkgolic acid as a well-known SUMO inhibitor. These results were in accordance with our previous findings indicating the SUMO inhibitory characteristics of curcumin [10, 15]. The SUMO inhibitory effect of Curcuma was reported by other authors as well. Hendriks et al. [55] demonstrated that curcumin was able to prevent the SUMOylation of histone H3. Also, more recently, it was shown to block the SUMOylation of RAD52, a protein important for DNA double-strand break repair [56]. Being SUMOylation activated and influenced by oxidative stress [13], these results, together with our previous findings, indicate that Curcuma could act as a SUMOylation inhibitor with both direct inhibiting protein SUMOylation and indirect inhibiting oxidative stress mechanisms.

The crosstalk between the SUMO and ubiquitin pathways was well known before [31]. In this regard, previously, we showed that oxidative stress induces SUMOylation while reducing ubiquitination [10]. Here, we found that the SUMO-1/ubiquitin ratio significantly increased in the control group indicating that the imbalance condition occurs probably due to an increase in oxidative stress condition. On the other hand, curcuma treatment was able to maintain the balance between these two dynamic processes and kept this balance at the healthy group level.

The antioxidant effect of Curcuma extract and its effect on SUMOylation, in addition to its JNK inhibitory characteristics on the retina in this study, were confirmed by our previous cellular studies in which we found that curcumin exerted an antioxidant effect and protected cells through modulating the SUMO-1-JNK axis [10].

## 5. Conclusion

**Figure 8:**
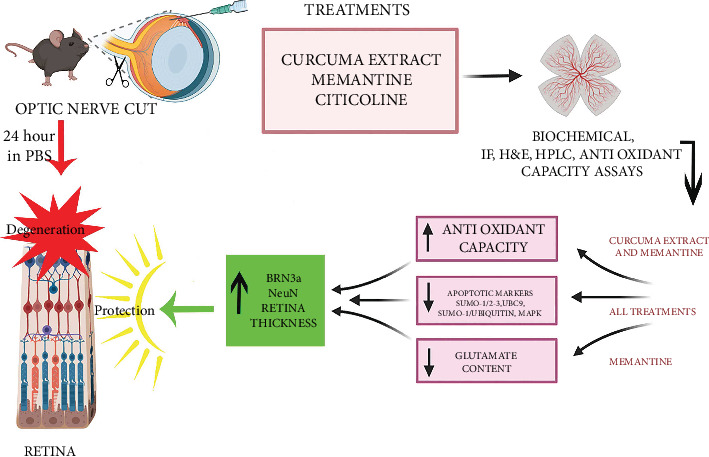
Summary cartoon of the obtained results. Representative flowchart cartoon of the obtained results showing how the retinal degeneration mouse model has been done and how the treatments were done for the 3 molecules used. The cartoon shows that three treatments have a different impact on the biological response in the retinal degeneration mouse model.

In conclusion, this study shed light on the mechanism of action of Curcuma, the main ingredient in turmeric, in retina protection. The antioxidant capacity of Curcuma and targeting SUMOylation seem to be important mechanisms in this *ex vivo* model of retinal degeneration (Figure 8). Clarifying the mechanism of action of Curcumin may help scientists to use this compound in addition to other protective agents to take advantage of additive or synergistic effects in therapeutic strategies.

## Data Availability

All data presented in this paper have been included in the manuscript. Data was collected in cells and retinal tissue assays and used for statistical analysis.

## Abbreviations

Cur: Curcuma extract
ONC: Optic nerve cut
RGCs: Retinal ganglion cells
Mem: Memantine
Cit: Citicoline
GA: Ginkgolic acid
SUMO: Small ubiquitin-related modifier.

## Supplementary Materials

Supplementary 1 Supplementary Figure 1: standard curve for the antioxidant capacity test for the Trolox assay kit. This curve has been used to assay the antioxidant capacities in the retinas after treatments.

Supplementary 2 Supplementary Figure 2: standard curve for the glutamate concentration. This curve has been used to assay the glutamate concentration tested on the retinas after treatments.

## References

[1] Wert K. J., Lin J. H., Tsang S. H., Casaroli-Marano R. P. (2014). General Pathophysiology in Retinal Degeneration. *Cell-Based Therapy for Retinal Degenerative Disease*.

[2] Morgan J. E. (2012). Retina ganglion cell degeneration in glaucoma: an opportunity missed? A review. *Clinical & Experimental Ophthalmology*.

[3] Tisi A., Feligioni M., Passacantando M., Ciancaglini M., Maccarone R. (2021). The impact of oxidative stress on blood-retinal barrier physiology in age-related macular degeneration. *Cells*.

[4] Nucci C., Martucci A., Giannini C., Morrone L. A., Bagetta G., Mancino R. (2018). Neuroprotective agents in the management of glaucoma. *Eye*.

[5] Thomas C. N., Berry M., Logan A., Blanch R. J., Ahmed Z. (2017). Caspases in retinal ganglion cell death and axon regeneration. *Cell Death Discovery*.

[6] Cheung W., Guo L. I., Cordeiro M. F. (2008). Neuroprotection in glaucoma: drug-based approaches. *Optometry and Vision Science*.

[7] Russo R., Cavaliere F., Berliocchi L. (2008). Modulation of pro-survival and death-associated pathways under retinal ischemia/reperfusion: effects of NMDA receptor blockade. *Journal of Neurochemistry*.

[8] Schmidt M., Giessl A., Laufs T., Hankeln T., Wolfrum U., Burmester T. (2003). How Does the Eye Breathe?:. *The Journal of Biological Chemistry*.

[9] Masuda T., Shimazawa M., Hara H. (2017). Retinal diseases associated with oxidative stress and the effects of a free radical scavenger (edaravone). *Oxidative Medicine and Cellular Longevity*.

[10] Buccarello L., Dragotto J., Iorio F., Hassanzadeh K., Corbo M., Feligioni M. (2020). The pivotal role of SUMO-1-JNK-Tau axis in an in vitro model of oxidative stress counteracted by the protective effect of curcumin. *Biochemical Pharmacology*.

[11] Marcelli S., Corbo M., Iannuzzi F. (2018). The involvement of post-translational modifications in Alzheimer’s disease. *Current Alzheimer Research*.

[12] Marcelli S., Ficulle E., Piccolo L., Corbo M., Feligioni M. (2018). An overview of the possible therapeutic role of SUMOylation in the treatment of Alzheimer's disease. *Pharmacological Research*.

[13] Feligioni M., Nisticò R. (2013). SUMO: a (oxidative) stressed protein. *Neuromolecular Medicine*.

[14] Sun Q., Qing W., Qi R. (2019). Inhibition of Sumoylation alleviates oxidative stress-induced retinal pigment epithelial cell senescence and represses proinflammatory gene expression. *Current Molecular Medicine*.

[15] Buccarello L., Dragotto J., Hassanzadeh K., Maccarone R., Corbo M., Feligioni M. (2021). Retinal ganglion cell loss in an ex vivo mouse model of optic nerve cut is prevented by curcumin treatment. *Cell Death Discovery*.

[16] Radomska-Leśniewska D. M., Osiecka-Iwan A., Hyc A., Góźdź A., Dąbrowska A. M., Skopiński P. (2019). Therapeutic potential of curcumin in eye diseases. *Central European Journal of Immunology*.

[17] Gordon O. N., Luis P. B., Ashley R. E., Osheroff N., Schneider C. (2015). Oxidative transformation of demethoxy- and bisdemethoxycurcumin: products, mechanism of formation, and poisoning of human topoisomerase II*α*. *Chemical Research in Toxicology*.

[18] Hassanzadeh K., Buccarello L., Dragotto J., Mohammadi A., Corbo M., Feligioni M. (2020). Obstacles against the marketing of curcumin as a drug. *International Journal of Molecular Sciences*.

[19] Ke Z., Zhang X., Cao Z. (2016). Drug discovery of neurodegenerative disease through network pharmacology approach in herbs. *Biomedicine & Pharmacotherapy*.

[20] Burugula B., Ganesh B. S., Chintala S. K. (2011). Curcumin attenuates staurosporine-mediated death of retinal ganglion cells. *Investigative Ophthalmology and Visual Science*.

[21] Kowluru R. A., Kanwar M. (2007). Effects of curcumin on retinal oxidative stress and inflammation in diabetes. *Nutrition and Metabolism*.

[22] Mansoor S., Gupta N., Luczy-Bachman G., Limb G. A., Kuppermann B. D., Kenney M. C. (2010). Protective effects of memantine and epicatechin on catechol-induced toxicity on Muller cells in vitro. *Toxicology*.

[23] Matteucci A., Varano M., Gaddini L. (2014). Neuroprotective effects of citicoline in in vitro models of retinal neurodegeneration. *International Journal of Molecular Sciences*.

[24] Brackett C. M., García-Casas A., Castillo-Lluva S., Blagg B. S. J. (2020). Synthesis and evaluation of ginkgolic acid derivatives as SUMOylation inhibitors. *ACS Medicinal Chemistry Letters*.

[25] Toklu H. Z., Scarpace P. J., Sakarya Y. (2017). Intracerebroventricular tempol administration in older rats reduces oxidative stress in the hypothalamus but does not change STAT3 signalling or SIRT1/AMPK pathway. *Applied Physiology, Nutrition, and Metabolism*.

[26] Fischer A. H., Jacobson K. A., Rose J., Zeller R. (2008). Hematoxylin and eosin staining of tissue and cell sections. *Cold Spring Harbor Protocols*.

[27] Mead B., Thompson A., Scheven B. A., Logan A., Berry M., Leadbeater W. (2014). Comparative evaluation of methods for estimating retinal ganglion cell loss in retinal sections and wholemounts. *PLoS One*.

[28] da Silva Moraes E. R., Grisolia A. B. A., Oliveira K. R. M. (2012). Determination of glutamate uptake by high performance liquid chromatography (HPLC) in preparations of retinal tissue. *Journal of Chromatography B*.

[29] Ito Y., Nakamura S., Tanaka H., Shimazawa M., Araie M., Hara H. (2008). Memantine protects against secondary neuronal degeneration in lateral geniculate nucleus and superior colliculus after retinal damage in mice. *CNS Neuroscience & Therapeutics*.

[30] Fukuda I., Ito A., Hirai G. (2009). Ginkgolic acid inhibits protein SUMOylation by blocking formation of the E1-SUMO intermediate. *Chemistry & Biology*.

[31] Lamoliatte F., McManus F. P., Maarifi G., Chelbi-Alix M. K., Thibault P. (2017). Uncovering the SUMOylation and ubiquitylation crosstalk in human cells using sequential peptide immunopurification. *Nature Communications*.

[32] Adibhatla R. M., Hatcher J. F. (2002). Citicoline mechanisms and clinical efficacy in cerebral ischemia. *Journal of Neuroscience Research*.

[33] Oshitari T., Fujimoto N., Adachi-Usami E. (2002). Citicoline has a protective effect on damaged retinal ganglion cells in mouse culture retina. *Neuroreport*.

[34] Schuettauf F., Rejdak R., Thaler S. (2006). Citicoline and lithium rescue retinal ganglion cells following partial optic nerve crush in the rat. *Experimental Eye Research*.

[35] Celiker H., Yuksel N., Solakoglu S., Karabas L., Aktar F., Caglar Y. (2016). Neuroprotective effects of memantine in the retina of glaucomatous rats: an electron microscopic study. *Journal of Ophthalmic and Vision Research*.

[36] Sasaoka M., Ota T., Kageyama M. (2020). Rotenone-induced inner retinal degeneration via presynaptic activation of voltage-dependent sodium and L-type calcium channels in rats.

[37] Weinreb R. N., Liebmann J. M., Cioffi G. A. (2018). Oral memantine for the treatment of glaucoma: design and results of 2 randomized, placebo-controlled, phase 3 studies. *Ophthalmology*.

[38] Chan W.-H., Wu H.-J., Hsuuw Y.-D. (2005). Curcumin inhibits ROS formation and apoptosis in methylglyoxal-treated human hepatoma G2 cells. *Annals of the New York Academy of Sciences*.

[39] Peng Y., Pu J., Tang C., Wu Z. (2017). Curcumin inhibits heat-induced apoptosis by suppressing NADPH oxidase 2 and activating the Akt/mTOR signaling pathway in bronchial epithelial cells. *Cellular Physiology and Biochemistry*.

[40] Sarawi W. S., Alhusaini A. M., Fadda L. M. (2021). Curcumin and nano-curcumin mitigate copper neurotoxicity by modulating oxidative stress, inflammation, and Akt/GSK-3*β* signaling. *Molecules*.

[41] Peddada K. V., Brown A., Verma V., Nebbioso M. (2019). Therapeutic potential of curcumin in major retinal pathologies. *International Ophthalmology*.

[42] Papastefanou V. P., Cohen V. M. L. (2011). Uveal melanoma. *Journal of Skin Cancer*.

[43] Vuong L., Conley S. M., Al-Ubaidi M. R. (2012). Expression and role of p 53 in the retina. *Investigative Ophthalmology & Visual Science*.

[44] Lu S., Zhao H., Zhou Y., Xu F. (2021). Curcumin affects leptin-induced expression of methionine adenosyltransferase 2A in hepatic stellate cells by inhibition of JNK signaling. *Pharmacology*.

[45] Chen Y., Yuan F., Lin J., Zhang X., Luo J., Huang L. (2021). Curcumin promotes the proliferation, invasion of neural stem cells and formation of neurospheres via activating SDF-1/CXCR4 axis. *Folia Neuropathologica*.

[46] Christensen I., Lu B., Yang N., Huang K., Wang P., Tian N. (2019). The susceptibility of retinal ganglion cells to glutamatergic excitotoxicity is type-specific. *Frontiers in Neuroscience*.

[47] Osborne N. N., Chidlow G., Layton C. J., Wood J. P. M., Casson R. J., Melena J. (2004). Optic nerve and neuroprotection strategies. *Eye*.

[48] Muangnoi C., Sharif U., Bhuket P. R. N., Rojsitthisak P., Paraoan L. (2019). Protective effects of curcumin ester prodrug, curcumin diethyl disuccinate against H_2_O_2_-induced oxidative stress in human retinal pigment epithelial cells: potential therapeutic avenues for age-related macular degeneration. *International Journal of Molecular Sciences*.

[49] López-Malo D., Villarón-Casares C. A., Alarcón-Jiménez J. (2020). Curcumin as a therapeutic option in retinal diseases. *Antioxidants*.

[50] Razavi B. M., Hosseinzadeh H. (2020). Antioxidant effects of *Curcuma longa* and its active constituent, curcumin, for the therapy of neurological disorders. *Oxidative Stress and Dietary Antioxidants in Neurological Diseases*.

[51] Cole G. M., Teter B., Frautschy S. A. (2007). Neuroprotective effects of curcumin. *Advances in Experimental Medicine and Biology*.

[52] Osawa T., Sugiyama Y., Inayoshi M., Kawakishi S. (1995). Antioxidative activity of tetrahydrocurcuminoids. *Bioscience, Biotechnology, and Biochemistry*.

[53] Ahsan H., Parveen N., Khan N. U., Hadi S. M. (1999). Pro-oxidant, anti-oxidant and cleavage activities on DNA of curcumin and its derivatives demethoxycurcumin and bisdemethoxycurcumin. *Chemico-Biological Interactions*.

[54] Kim D., Maharjan P., Jin M. (2019). Potential albumin-based antioxidant nanoformulations for ocular protection against oxidative stress. *Pharmaceutics*.

[55] Hendriks I. A., D’Souza R. C. J., Yang B., Vries M. V.-d., Mann M., Vertegaal A. C. O. (2014). Uncovering global SUMOylation signaling networks in a site-specific manner.

[56] Tseng W. C., Chen C. Y., Chern C. Y. (2021). Targeting HR repair as a synthetic lethal approach to increase DNA damage sensitivity by a rad52 inhibitor in brca2-deficient cancer cells. *International Journal of Molecular Sciences*.

