# Supplementary figures and images for: Protective Effect of Curcuma Extract in an Ex Vivo Model of Retinal Degeneration via Antioxidant Activity and Targeting the SUMOylation
Source: Oxid Med Cell Longev. 2022 Jul 29;2022:8923615. doi: 10.1155/2022/8923615 (PMC9356244; doi:10.1155/2022/8923615)

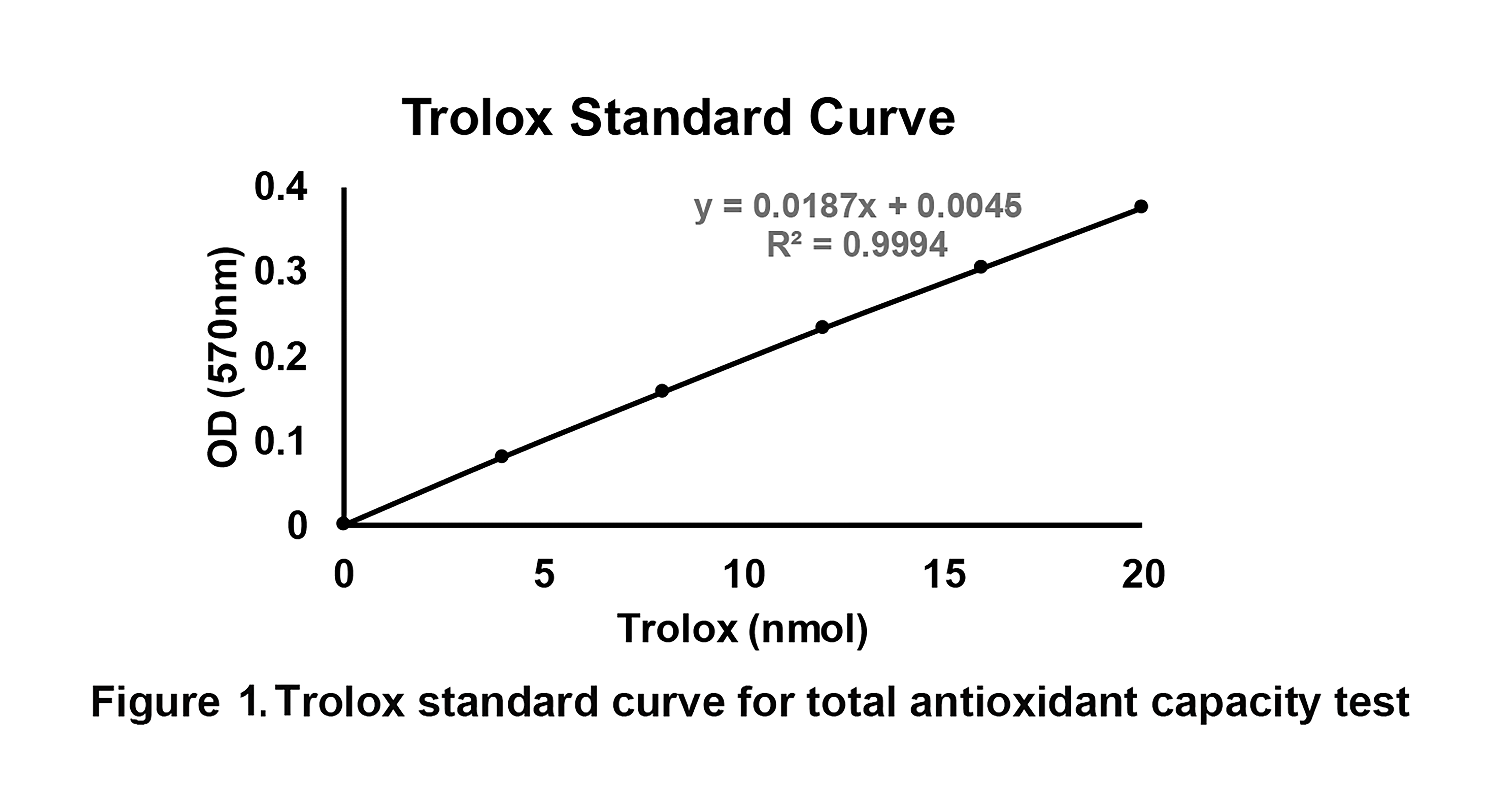

Supplement: Supplementary 1 — Supplementary Figure 1: standard curve for the antioxidant capacity test for the Trolox assay kit. This curve has been used to assay the antioxidant capacities in the retinas after treatments. [file 8923615.f1.doc]

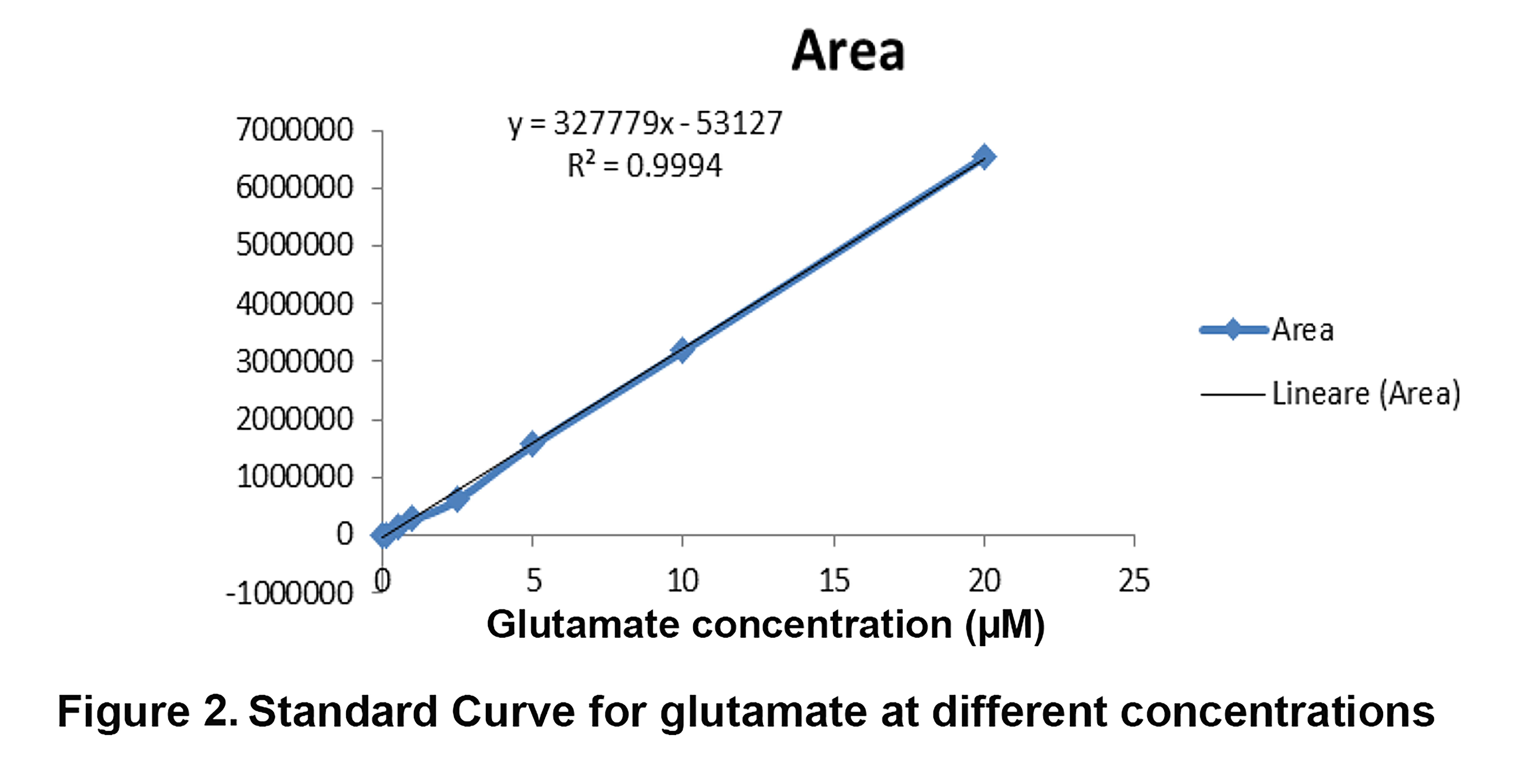

Supplement: Supplementary 2 — Supplementary Figure 2: standard curve for the glutamate concentration. This curve has been used to assay the glutamate concentration tested on the retinas after treatments. [file 8923615.f2.doc]
